# Management of Renal Failure and Ascites in Patients with Cirrhosis

**DOI:** 10.4061/2011/790232

**Published:** 2011-09-27

**Authors:** Kaushal Madan, Ashish Mehta

**Affiliations:** Medanta Institute of Digestive and Hepatobiliary Sciences, Medanta-The Medicity Hospital, Sector 38, Gurgaon, Haryana 122001, India

## Abstract

Ascites and renal dysfunction in cirrhosis occur when the liver disease is decompensated and signify the presence of advanced liver failure. However, the precipitating causes should be looked for and treated. Although liver transplantation is the treatment of choice in patients with advanced liver failure, mild to moderate ascites can be treated effectively with medical management. Similarly, renal failure in cirrhotics is reversible if the precipitating causes can be treated effectively and by use of combination of vasoconstrictors and albumin. Transjugular intrahepatic portosystemic shunts also offer an effective therapy for refractory ascites and HRS. Such treatments may offer effective bridge to liver transplantation, by improving short and medium term survivals. Here, we shall discuss all the options available for the management of these complications of cirrhosis.

## 1. Introduction

Ascites is one of the indicators of decompensation and poor prognosis in patients with cirrhosis of any etiology. Once ascites develops, the predicted mortality is approximately 50% at 2 years [1]. Ascites is also an indicator of advanced portal hypertension. In many natural history series of cirrhosis, ascites is the most frequent first complication of cirrhosis preceded only by hepatocellular carcinoma [2]. In addition to being a poor prognostic factor, it also leads to significant morbidity in cirrhotics. But it is important to remember that patients with cirrhosis are not immune to develop ascites due to other causes, such as tuberculosis, malignancy, intrinsic renal disease, or heart failure. For this reason, it is important to carry out a complete evaluation and treat it appropriately. 

## 2. Diagnosis of Ascites

When a cirrhotic presents for the first time with abdominal distension then, unless proved otherwise, the ascites is secondary to portal hypertension. A reasonable estimate can be made from a detailed history, examination, and biochemical assessment. 

### 2.1. Ascitic Fluid Analysis

A detailed laboratory assessment of the ascitic fluid is a must in all patients who present with ascites for the first time. It confirms the diagnosis of cirrhotic ascites, rules out other causes of ascites, and also detects presence of spontaneous bacterial peritonitis. Measurement of serum to ascitic fluid albumin gradient (SAAG) readily differentiates ascites due to portal hypertension and ascites due to other causes. Almost simultaneous measurement of ascitic fluid and serum albumin is required. SAAG of ≥1.1 suggests the presence of portal hypertension with an accuracy of 97% [3]. The importance of measuring total ascitic fluid protein is to assess the risk of developing spontaneous bacterial peritonitis (SBP) later and therefore recommending antibiotics for primary prophylaxis of SBP. Cirrhotics who have total ascitic fluid protein concentration less than 1.5 gm/dL are at an increased risk of developing SBP [4]. Measurement of total and differential cell count is essential at both the initial evaluation and all subsequent times when ascitic fluid is drained, in order to look for evidence of SBP. SBP is diagnosed when the neutrophil count of the ascitic fluid is more than 250/cumm. The prevalence of SBP in cirrhotic patients attending the outpatient clinics is 1.5–3.5% [5]. At the same time ascitic fluid should be sent for culture by inoculating in the blood culture bottles bed side. This technique can yield a positive culture in about 40% of the cases.

Patients who have high ascitic fluid protein content along with lymphocyte predominant ascites usually have other inflammatory or malignant causes for ascites, such as peritoneal tuberculosis [6] or peritoneal metastatic deposits. Adenosine deaminase enzyme which is released from lymphocytes has been shown to be raised in patients with peritoneal tuberculosis. In a meta-analysis of 4 studies which included 264 patients, peritoneal fluid ADA had a sensitivity of 100% and specificity of 97% for making a diagnosis of tubercular ascites. The optimal cut-off value defined was 39 IU/L [7]. In another article, the ADA among patients with tubercular ascites was found to be significantly higher than the values in patients with other causes of ascites (septic peritonitis, malignant ascites, and transudative ascites) [8].

## 3. Ascites and Its Management

Ascites in cirrhotics should be treated because it is associated with discomfort, reduced respiratory excursion, reduced appetite because of pressure effect, and predisposition to SBP. Presence of current ascites also negatively impacts the quality-of-life scores in cirrhotics and therefore warrants treatment [9]. For management purposes, ascites has be classified into mild or grade 1 (only detectable by ultrasonography), moderate or grade 2 (moderate symmetrical distension of abdomen), and severe or grade 3 (large or tense ascites), by the international ascites club.

### 3.1. Salt Restriction

Dietary salt restriction should be recommended for all patients who present with ascites for the first time and have grade 1 or 2 ascites. The recommended salt intake in such patients is between 80–120 mmol of sodium per day, which corresponds to 4.6–6.9 gm of salt. A negative sodium balance can be obtained by reducing dietary salt intake in 10–20% of cirrhotics with ascites [10]. 

### 3.2. Diuretics

In the initial management of mild to moderate ascites (which is not tense), aldosterone antagonists should be started first, since the pathophysiology of sodium retention in cirrhotics is due to increased reabsorption of sodium from the proximal and distal tubule and the mediator of this reabsorption is secondary hyperaldosteronism [11]. Spironolactone, which is an aldosterone antagonist, should be started first in a dose of 100 mg/day and the dose increased in 100 mg increment every 7 days till 400 mg. Beyond this, loop diuretic, furosemide should be added in a dose of 40 mg per day and added in increment of 40 mg till a total of 160 mg. The dose of diuretics should be adjusted to achieve a weight reduction of 0.5 kg/day in patients without pedal edema and about 1.0 kg/day in patients with pedal edema. Higher doses of diuretics in patients without pedal edema can result in complications such as hyponatremia or azotemia. It has been suggested that for patients with mild to moderate ascites who present for the first time, the above mentioned regimen should be followed, but for patients with resistant ascites or recurrent ascites, a combination of spironolactone and furosemide (100 mg and 40 mg, resp.) should be started at the outset [12]. The newer loop diuretic torsemide is more potent than furosemide and has been shown to be as effective and safe as furosemide in a small study of 46 cirrhotics with ascites [13].

Diuretics can induce electrolyte imbalances; furosemide can induce hypokalemia, spironolactone can induce hyperkalemia because of its potassium sparing effect, and both these diuretics can induce hyponatremia. Therefore, furosemide should be discontinued if serum potassium is <3 mmol/L, and spironolactone should be stopped if serum potassium is >6 mmol/L. If the serum sodium is <120 mmol/L, no diuretic should be given. Diuretics should also be discontinued if there are other diuretic induced complications such as renal failure, worsening hepatic encephalopathy, or severe muscle cramps.

### 3.3. Large Volume Paracentesis (LVP)

LVP, as the name suggests, is defined as drainage of large volumes (>5-6 litres) of ascites. It is the treatment of choice for tense ascites (grade 3 ascites). It is more effective and safer than just diuretic therapy for tense ascites. But diuretics should always be given after LVP in order to prevent reaccumulation of ascites, since diuretics would be required to reverse the pathophysiology of sodium retention. 

However, LVP may be associated with the development of post-paracentesis circulatory dysfunction (PPCD) which involves a rise in cardiac output, fall in systemic vascular resistance, and a rise in serum rennin and aldosterone. These changes are usually maintained for up to 24 hours, and the hormonal changes may last up to 6 days [14]. PPCD can be prevented by concomitant administration of plasma expanders, and the most effective plasma expander for this purpose has been demonstrated to be albumin which should be given in a dose of 8 gm/litre of ascitic fluid drained. Although cheaper alternatives such as dextran-70 have also been used effectively to prevent PPCD associated with LVP [15], albumin has been shown to be more effective than other plasma expanders if volumes of >5 litres are removed. In this randomized controlled trial, the incidence of PPCD was 18.5%, 34.4%, and 37.85 in patients receiving albumin, dextran-70, and polygeline, respectively, and the type of plasma expander used has been shown to be an independent predictor of development of PPCD [16, 17].

LVP is also an effective treatment for refractory ascites. Refractory ascites can be divided into two categories: diuretic-resistant ascites (defined as the ascites that cannot be mobilized, or early recurrence of which cannot be prevented due to lack of response to adequate sodium restriction and diuretic treatment; patients should be taking at least 400 mg of spironolactone and 160 mg of furosemide for at least one week, along with salt restricted diet of <90 mmol/L) and diuretic-intractable ascites (defined as the ascites that cannot be mobilized, or early recurrence of which cannot be prevented because of development of complications of diuretic dose such as, diuretic-induced hepatic encephalopathy, renal dysfunction, hyponatremia, hypo- or hyperkalemia) [18]. 

### 3.4. Transjugular Intrahepatic Portosystemic Shunts (TIPS)

Since ascites in cirrhosis develops due to portal hypertension, it would seem logical to decompress the portal system to reduce the ascites. So TIPS has been tried in several uncontrolled and controlled trials for refractory ascites. TIPS is useful and safe in patients with refractory ascites, where portal hypertension is not associated with presence of advanced liver failure. The randomized trials which assessed the role of TIPS versus LVP had excluded patients who had evidence of advanced liver disease (serum bilirubin > 5 mg%, INR > 2, presence of recurrent or persistent hepatic encephalopathy, renal failure). These trials consistently showed better control of ascites with TIPS, but the effect on survival was inconsistent. The studies that included small number of patients or included a mix of refractory and recurrent ascites did show some survival advantage [19–21], but the studies which included purely refractory ascites and had significant sample sizes did not show any survival advantage of TIPS over LVP [22, 23]. Meta-analysis including these five trials (>300 patients) again demonstrated that there was significantly better control of ascites (OR ranging from 0.07–0.56) with a higher incidence of hepatic encephalopathy (OR ranging from 1.72 to 2.26) in the TIPS group [24–27], and only one meta-analysis demonstrated an increase in transplant-free survival in patients undergoing TIPS (*P* = 0.035) [28]. TIPS appears to be an effective therapy for refractory ascites, but it should be emphasized that the patients should be carefully selected for this procedure. 

### 3.5. Aquaretics

Since the basic pathophysiology of water retention and dilutional hyponatremia in cirrhotics is antidiuretic hormone or arginine vasopressin (AVP) induced water resorption from the distal collecting duct, it would appear logical to block this action of AVP and inhibit the pure water resorption. AVP acts at this level through the V2 receptors on the distal collecting tubule. Recently, a new class of drugs called vaptans, which act by blocking the V2 receptors have been shown to improve free water clearance in patients with a number of conditions associated with water retention, such as congestive heart failure and cirrhosis.

Initial studies with an orally active V2 receptor blocker, satavaptan, did show improvement in hyponatremia and control of ascites in combination with diuretics, but a phase 3 RCT in combination with diuretics failed to demonstrate a significant effect on control of ascites. In addition, there was an increase in morbidity and mortality in the active treatment arm [29]. Recently, another V2 receptor blocker, tolvaptan, has been approved for management of dilutional hyponatremia in cirrhotics [30] and is expected to help in reduction of water retention as well in these patients. 

### 3.6. Liver Transplantation

All patients with refractory ascites have advanced liver failure and therefore should be offered liver transplantation, if all other precipitating causes of acute deterioration have been ruled out. However, many patients who have ascites may not meet the MELD score cutoffs where transplantation is recommended. MELD score, alone, probably underestimates the risk of mortality in patients who have ascites [31].

## 4. Renal Failure in Cirrhosis and Its Management

Renal dysfunction among cirrhotics is associated with a very poor prognosis, so it forms a part of the prognostic MELD score. Acute renal dysfunction or acute kidney injury (AKI) (abrupt rise in serum creatinine by 0.3 mg%) in cirrhotics can be classified into prerenal azotemia (volume responsive prerenal AKI), acute tubular necrosis (ATN) and hepatorenal syndrome (HRS) (volume unresponsive prerenal, functional type AKI). In an Indian tertiary care hospital, the most common cause of AKI in cirrhotics was found to be acute tubular necrosis (44.4%), followed by prerenal azotemia (36.4%), and hepatorenal syndrome (HRS) (19.2%) [32]. However, studies from the west indicate that the most common form of AKI among cirrhotics is prerenal (volume responsive) azotemia (66%) followed by ATN and HRS being the least common form [33]. Here, we shall discuss the management of HRS in cirrhotics since it is the most severe and prognostically most important form of renal failure in this group of patients.

Other forms of renal failure can be differentiated from HRS, in cirrhotics, by urine routine and microscopic examination (presence of significant proteinuria, casts and/or hematuria suggests intrinsic renal disease), ultrasound examination of kidney, ureters and bladder (presence of shrunken kidneys with loss of corticomedullary differentiation or presence of obstructive uropathy suggests non-HRS AKI), response to fluid replacement (improvement in serum creatinine with volume replacement suggests prerenal AKI), and by history of recent use of nephrotoxic drugs and active sepsis (suggest acute tubular necrosis). The principles of management of non-HRS AKI depend on the cause of AKI. However, when it is difficult to rule out other causes, it is important to replace volume as is described below; stop all nephrotoxic drugs, and treat active sepsis if present. This would take care of most forms of renal failure. Dialysis may be required for specific indications (hyperkalemia, metabolic acidosis, uremic encephalopathy, and pericarditis).

## 5. Hepatorenal Syndrome

HRS is defined as the development of renal failure in patients with advanced liver disease in the absence of other identifiable causes of renal failure. Recently, modified criteria have been laid down for the diagnosis of hepatorenal syndrome (Table 1). So it is important to exclude hypovolemia, use of nephrotoxic drugs, and presence of intrinsic renal disease before a diagnosis of HRS can be made. One of the important changes from the previous definition of HRS is the understanding that HRS can also be diagnosed in the presence of active sepsis, which earlier used to be an exclusion criteria. HRS can be of two types. Type 1 HRS develops rapidly with a rise in serum creatinine to >2.5 mg% in less than 2 weeks. It is usually preceded by a precipitating event, and the most common being some bacterial infection such as spontaneous bacterial peritonitis. Type 2 HRS is characterized by a slower development of renal dysfunction and usually develops in the setting of refractory ascites. According to another recent, modified classification of renal failure among cirrhotics, given by a working party, type 1 HRS may be considered as a form of AKI in cirrhotics and type 2 HRS may be considered as CKD in cirrhotics [34]. HRS type I has a very poor prognosis in cirrhotics and predicts a median survival of only 3 months [35], and untreated type 1 HRS has a median survival of about 1 month. 

## 6. Treatment of HRS (Table 2)

As has been mentioned earlier, hypovolemia needs to be corrected by stopping all diuretics for at least 48 hours and by administration of albumin before labelling a patient as having HRS. Sepsis should be actively looked for (since sepsis is the most common precipitant of HRS), by blood cultures, urine cultures, ascitic fluid cytology and cultures, and chest radiographs, and treated with appropriate antibiotics. 

### 6.1. Vasoconstrictors

Vasoconstrictors act by counteracting the strong splanchnic vasodilatation, which is characteristic of advanced cirrhosis. The most common drug used for this purpose is the vasopressin analogue, terlipressin. Terlipressin is used at a dose of 1mg every 4–6 hrly and increased, if there is no response (<25% reduction in serum creatinine at day 3), to a maximum of 2 mg every 4–6 hourly”. Treatment is to be continued till the serum creatinine falls to less than 1.5 mg%. Trial of treatment with terlipressin should be continued up to 2 weeks. Beyond this, if there is no response, it should be discontinued. This has to be given along with albumin in a dose of 1 gm/kg on day 1 followed by 20–40 gm per day. Treatment with terlipressin is associated with improvement in urine output, reduction in creatinine levels, reduction in renin levels, and improvement in mean arterial pressure. Effect on survival was demonstrable in some but not all studies. A systemic review of the use of vasoconstrictors in patients with type 1 and type 2 HRS demonstrated that terlipressin plus albumin improved short term survival (15 days survival) (RR 0.81, 0.68–0.97) in patients with type 1 HRS, but not in type 2 HRS. There was no improvement in 30-day, 90-day, or 180-day survival [36]. Another systematic review, which included 4 RCTs of terlipressin in type 1 HRS, demonstrated reversal of HRS and trend towards improved 90 days survival [37]. Use of terlipressin is associated with ischemic side effects (cardiac, digital, and mesenteric) in as many as 12% of patients, and it is usually contraindicated in patients who have coronary artery disease and peripheral vascular disease.

Noradrenaline infusion (dose ranging from 0.5 to 3 mg/hour) along with albumin has also been shown to be as effective as terlipressin plus albumin in improving renal function and circulatory function in patients with HRS [38]. Another larger open lablelled RCT (20 patients in each arm; noradrenaline plus albumin versus terlipressin plus albumin), published from India, demonstrated similar improvement in renal functions and similar survival rates in the two groups [39]. 

Alpha-adrenergic agonist, midodrine, is another vasoconstrictor which has been used in patients with HRS. Midodrine was used in a dose of 2.5 to 12.5 mg orally every 8 hourly in combination with octreotide 100 *μ*g subcutaneously, 8 hourly in 5 patients with HRS type 1. These were compared with 8 patients who were managed with standard therapy. Both groups also received albumin (50–100 mL daily). Patients who received the combination therapy had reversal of HRS with significant increase in GFR and reduction in plasma renin activity. There were no ischemic side effects [40]. 

### 6.2. Transjugular Intrahepatic Portosystemic Shunts (TIPS) for HRS

TIPS has been used to control portal hypertension and has been found to be useful in patients with HRS as well. However, many patients with advanced liver disease with renal failure have contraindications for the use of TIPS. A single centre study in 129 patients with long-term followup demonstrated a significant improvement in creatinine values after placement of TIPS. Amount of iodinated contrast medium administered did not affect creatinine levels [41]. Among 41 patients (21 with HRS type 1 and 20 with HRS type 2), TIPS placement not only improved creatinine clearance (18 ± 15 to 48 ± 42 mL/min) and urinary sodium excretion (9 ± 16 to 77 ± 78 mmol/24 hours), but also gave a one-year survival of 48% [42]. However, there is no RCT comparing TIPS with other forms of therapy in patients with HRS.

### 6.3. Albumin Dialysis

Albumin dialysis is supposed to act on the principle of removing albumin bound toxins, which in case of HRS would be cytokines and vasodilators. In a small RCT among 13 patients with type 1 HRS, there was a significant improvement in renal function and short-term mortality (100% at day 7 in the standard medical therapy group (*n* = 5) versus 26.5% in the MARS group (*n* = 8)) in patients undergoing molecular adsorbent and recirculating system (MARS) therapy [43]. However, a recent pilot study in 6 patients with HRS who had failed therapy with vasoconstrictors could not demonstrate any benefit of this therapy, either on systemic hemodynamics or on survival [44].

### 6.4. Liver Transplantation

Patients with HRS have advanced liver failure and therefore qualify to undergo liver transplantation. Over all, long-term survival after liver transplantation has been reported to be around 65%. Presence of HRS, if sepsis is excluded, should be an indication for urgent/semiurgent liver transplantation. In such cases, other forms of therapy such as vasoconstrictors or albumin dialysis may be used as a bridge to transplantation. Although recent studies suggest that there is no difference in survival between patients with or without HRS (95% 1-year survival in presence of HRS versus 86% in its absence) [45], who are transplanted, it is always desirable to have the renal dysfunction corrected before a patient is taken up for transplantation. In a retrospective study, 9 patients with HRS were first treated with vasoconstrictors and then transplanted. These were compared with 27 patients without HRS who were also transplanted. The outcomes following transplantation were similar between the two groups with similar three-year survival probability (100% in treated HRS group versus 83% in the non-HRS group) [46]. So after reversal of HRS by vasoconstrictors, the patients should be listed for a semiurgent liver transplantation even if the serum creatinine has normalized.

## 7. Summary

Ascites and renal failure in cirrhotics suggest advanced portal hypertension and poor liver function and therefore predict poor prognosis. Ascites may be the first sign of progression of liver dysfunction or may even suggest an underlying complication such as development of a hepatocellular carcinoma. Mild or moderate ascites can usually be managed by salt restriction along with diuretics. For severe or tense ascites, large volume paracentesis with albumin infusion is required along with continued use of diuretics. For refractory ascites, the options are either repeated LVP plus albumin or TIPS. Renal failure in cirrhotics can be because of a number of causes, and HRS is not the most common cause of renal failure among cirrhotics. The most common cause is either volume responsive prerenal failure or acute tubular necrosis. Presence of HRS signifies advanced liver dysfunction, and ideal treatment is liver transplantation for such patients. But it is advisable to reverse HRS prior to transplantation. Treatment is initiated by excluding/treating precipitating causes such as SBP, correction of hypovolemia, and discontinuation of diuretics. Specific treatment involves the use of a combination of vasoconstrictors and albumin. Terlipressin has been shown to be effective in most cases, and noradrenaline has also been shown to be as effective as terlipressin. Another strategy which has been found to be effective is a combination of midodrine, octreotide, and albumin. TIPS has also been shown to be effective in improving renal failure in patients with HRS but should only be used as a bridge to liver transplantation. Finally, for both patients with ascites and HRS, the treatment of choice remains liver transplantation which corrects the basic pathophysiology of these two complications.

## Figures and Tables

**Table 1:** Diagnostic criteria for hepatorenal syndrome.

Modified criteria for diagnosis of hepatorenal syndrome
Cirrhosis with ascites
Serum creatinine > 1.5 mg%
Absence of shock
Absence of hypovolemia (no improvement in renal function after at least 2 days of diuretic withdrawal and volume expansion with albumin in a dose of 1 gm/kg/day)
No ongoing or recent treatment with nephrotoxic drugs
Absence of intrinsic renal disease (proteinuria < 0.5 gm/day; urine RBCs < 50/HPF; normal renal ultrasound)

**Table 2:** Therapeutic modalities used in HRS and their effect on renal function and survival.

Therapeutic modality	Studies	Improved renal function	Improved survival
Terlipressin plus albumin	RCTs and meta-analysis	Yes	Yes
Noradrenaline plus Albumin	RCTs	Yes	? yes
Midodrine plus octreotide plus albumin	Single small RCT	Yes	No
TIPS	Non-RCTs	Yes	No
Albumin dialysis	Small RCT	Yes	No
Liver transplantation		Yes	Yes
